# Cross-sectional relationship between haemoglobin concentration and measures of physical and cognitive function in an older rural South African population

**DOI:** 10.1136/jech-2018-210449

**Published:** 2018-04-21

**Authors:** Collin F Payne, Justine I Davies, F Xavier Gomez-Olive, Katherine J Hands, Kathleen Kahn, Lindsay C Kobayashi, Brent Tipping, Stephen M Tollman, Alisha Wade, Miles D Witham

**Affiliations:** 1 Center for Population and Development Studies, Harvard University, Boston, Massachusetts, USA; 2 School of Population Sciences and Health Services Research, Faculty of Life Sciences and Medicine, Centre for Global Health, King’s College London, London, UK; 3 Medical Research Council/Wits University Rural Public Health and Health Transitions Research Unit, Faculty of Health Sciences, School of Public Health, University of the Witwatersrand, Johannesburg, South Africa; 4 Scottish National Blood Transfusion Service, Ninewells Hospital, Dundee, South Africa; 5 Epidemiology and Global Health Unit, Department of Public Health and Clinical Medicine, Umeå University, Umea, Sweden; 6 INDEPTH Network, Accra, Ghana; 7 Lombardi Comprehensive Cancer Center, Georgetown University, Washington, District of Columbia, USA; 8 Division of Geriatric Medicine, School of Clinical Medicine, Faculty of Health Sciences, University of the Witwatersrand, Johannesburg, South Africa; 9 Ageing and Health, School of Medicine, University of Dundee, Dundee, UK

**Keywords:** ageing, functioning and disability, chronic di, international hlth, physical function

## Abstract

**Background:**

Age cohort differences in haemoglobin concentrations and associations with physical and cognitive performance among populations of lower income and middle-income countries have not previously been described. We examined the association between these factors among older men and women in rural South Africa.

**Methods:**

We analysed cross-sectional data from a population-based study of rural South African men and women aged 40 and over (n=4499), with data drawn from questionnaire responses, a cognitive battery, objective physical function tests and blood tests. Anaemia was defined as a haemoglobin concentration <12 g/dL for women and <13 g/dL for men. We related haemoglobin concentrations to each of age, grip strength, walk speed and a latent cognitive function z-score for men and women separately. We used unadjusted correlations and linear models to adjust for comorbidities and inflammation.

**Results:**

In total, 1042 (43.0%) women and 833 (40.1%) men were anaemic. Haemoglobin concentrations were inversely correlated with age for men but not for women; in adjusted analyses, haemoglobin was 0.3 g/dL lower per decade older for men (95% CI 0.2 to 0.4 g/dL). In adjusted analyses, haemoglobin concentration was independently associated with grip strength in women (B=0.391, 95% CI 0.177 to 0.605), but this did not reach significance in men (B=0.266, 95% CI −0.019 to 0.552); no associations were observed between haemoglobin levels and walk speed or cognitive score.

**Conclusions:**

Anaemia was prevalent in this study population of middle-aged and older, rural South African adults, but in contrast to high-income countries, it was not associated with poor physical or cognitive function. Our findings need to be replicated in other populations.

## Introduction

Anaemia is a complex phenomenon in older people. Mild anaemia, defined by the WHO as haemoglobin <13.0 g/dL in men and <12.0 g/dL in women,^1^ is common in older people in high-income countries (HICs), affecting between 10% and 25% of those aged 65 years and over.^2–4^ Even mild anaemia in older people is associated with impaired physical and cognitive function^5–7^ and with increased risks of hospitalisation and mortality.^3 8 9^ Although no trials have been completed to show improvements in physical and cognitive functions with treatment for anaemia, many physicians assume that treating anaemia will bring benefit in these domains. Previous work in South Africa has shown an estimated prevalence of anaemia of 17.5% in the general adult population, driven at least in part by high levels of iron deficiency.^10 11^

As the populations of older people living in lower and middle-income countries (LMICs) such as South Africa continue to grow in size, anaemia has the potential to cause a substantial yet potentially modifiable burden to individual and population health in these countries. However, most studies of anaemia in older people have been conducted in HICs. Answers to fundamental questions about anaemia among the older populations of LMICs, including its population-specific prevalence, causes and consequences will be critical for ensuring commensurate research, clinical and public health responses to anaemias. In HICs, common causes of anaemia in older people include iron deficiency caused by chronic gastrointestinal blood loss, vitamin B_12_ deficiency, anaemia of chronic disease driven by chronic inflammation, renal dysfunction and bone marrow dysfunction; these causes frequently coexist.^12 13^ In LMICs, additional common causes such as chronic infections like HIV, as well as malaria, parasitic infestations, malnutrition and haemoglobinopathies may add to the prevalence and severity of anaemia in older populations.^14^

It is also unclear whether the relationship between low haemoglobin and adverse outcomes such as impaired physical and cognitive performance is causal. While biologically plausible causal pathways can be posited, it is equally possible that low haemoglobin concentrations are a marker, rather than a causal factor, in explaining these associations. Such relationships have not been studied in older populations in LMICs,^14^ and before attempting to design and test healthcare pathways to investigate and treat low haemoglobin concentrations in older populations living in LMICs, a better understanding is required of the prevalence and consequences of anaemia in these populations. These data are important to define the scope of the problem, the target subpopulations most likely to benefit from intervention and the thresholds at which it would be appropriate to trigger intervention.

In this analysis, we used data from a population-based study of middle-aged and older adults in rural South Africa to address some of these questions. We aimed to investigate: (A) the prevalence of anaemia in middle-aged and older adults, overall and by sex and age group, (B) the associations between haemoglobin concentrations and physical and cognitive function; and (C) the degrees to which these associations may be explained by the presence of comorbid disease.

## Methods

### Population

We used data from the 2015 baseline wave of the ‘Health and Aging in Africa: A Longitudinal Study of an INDEPTH community in South Africa’ (HAALSI) survey of middle-aged and older people. The methods of this study have been described elsewhere.^15^ HAALSI was conducted during 2014–2015 in the Agincourt Health and Demographic Survey Site (HDSS), based in the Agincourt subdistrict of South Africa.^16^ The Agincourt site is a rural area of low socioeconomic status in the northeast of South Africa, near the Mozambique border, with a population of approximately 116 000 people living in 32 villages. It has a high proportion of immigrant and former refugee Mozambicans; the majority of the population in the Agincourt site are of Tsongan ethnicity. Participants in HAALSI were selected based on being permanently resident in the Agincourt HDSS area during the 12 months prior to the 2013 HDSS census round, and with a self-reported age of 40 years or over on 1 July 2014. A total of 6281 people were selected to be approached, and of these, 5059 (86%) agreed to take part in the main baseline HAALSI survey. No exclusion criteria beyond inability to provide informed consent were applied. The analysis sample for the analyses described in this paper was the 4499/5059 (89%) individuals who provided an analysable blood sample for haemoglobin concentrations. No other exclusion criteria were applied for this analysis. All participants gave informed consent to participate in the study, and the study was conducted according to the principles embodied in the Declaration of Helsinki.

### Outcome and covariate measurements

Haemoglobin was tested using capillary blood obtained from a fingerprick sample, tested on a Hemocue HB 201 analyser (Hemocue AB, Angelholm, Sweden). Anaemia was defined according to the current WHO and local South African reporting conventions: haemoglobin <13.0 g/dL for men, <12.0 g/dL for women^1 10^; moderately severe anaemia was defined as haemoglobin 8.0–10.9 g/dL for both sexes; and severe anaemia as haemoglobin <8.0 g/dL for both sexes. As this was a point of care test, no other information was available (eg, on erythrocyte size, morphology or mean red cell haemoglobin concentration). The Hemocue HB 201 system has previously shown acceptable agreement with laboratory haemoglobin measures, without systematic bias.^17^ Walk speed, grip strength, cognitive performance, comorbid disease and C reactive protein (CRP) levels were assessed; methods for the measurement of these variables are given in the online supplementary information.

10.1136/jech-2018-210449.supp1 Supplementary data

### Statistical analysis

Descriptive statistics were generated for baseline health and demographic variables from the full HAALSI cohort, as well as according to anaemia status. Both haemoglobin levels and physical performance measures vary between men and women; women have lower haemoglobin levels and lower muscle strength at all ages^3 18^; therefore, all analyses are done separately for men and women. The relationship between haemoglobin concentration and each of cognitive z-score, grip strength and walk speed was depicted graphically, as was the relationship between decade of age and mean haemoglobin concentration, both for the full analysis sample and for those individuals who were defined as healthy (those without impaired activities of daily living, without comorbidity (diabetes mellitus, hypertension, angina, stroke, HIV infection or chronic bronchitis) and with CRP <4 mg/L). Pearson’s correlation coefficient was calculated for the correlation between haemoglobin concentration and both age and cognitive z-score; Spearman’s rho was calculated for the correlation between haemoglobin concentration and both grip strength and walk speed. Haemoglobin concentrations in the presence or absence of each comorbid disease (diabetes mellitus, hypertension, angina, stroke, HIV infection and chronic bronchitis) were compared using Student’s t-test.

Linear regression was used to estimate the independent association between age (in years) and haemoglobin concentration, adjusting for logCRP, hypertension, diabetes mellitus, angina, stroke, HIV infection and chronic bronchitis. Linear regression was also used to estimate the independent association between haemoglobin concentration and latent cognitive z-score, adjusted for the above covariates, age (in years) and education level. Grip strength and walk speed were treated as continuous variables but were not normally distributed; the distribution approximated a Tweedie distribution with power 1.2. Generalised linear modelling using this distribution was used to estimate the associations between haemoglobin concentration and each of these physical performance measures, again adjusted for age (in years), logCRP and comorbidities as above. Analyses were run separately for each outcome measure, and cases with missing covariate data were excluded from multivariate analyses. Given the high prevalence of HIV in the study population, and the known strong relationship between HIV infection and low haemoglobin,^19^ subgroup analyses were performed on the group who were HIV positive. All analyses were performed using SPSS V.24, and a two-sided p value of <0.05 was taken as statistically significant for all analyses.

## Results

Characteristics of the sample, overall and according to anaemia status, are shown in table 1.

**Table 1:** Baseline details of analysis population (n=4499)

	All (n=5059)	Anaemic* (n=1875)	Not anaemic (n=2624)	No Hb measurement (n=560)
Mean age (years) (SD)	61.7 (13.1)	62.4 (13.3)	61.4 (12.8)†	60.9 (13.5)†
Age group (%)				
40–49	918 (18.1)	321 (17.1)	483 (18.4)	114 (20.4)
50–59	1410 (27.9)	496 (26.5)	746 (28.4)	168 (30.0)
60–69	1304 (25.8)	497 (26.5)	680 (25.9)	127 (22.7)
70–79	878 (17.4)	343 (18.3)	450 (17.1)	85 (15.2)
80+	549 (10.9)	218 (11.6)	265 (10.1)	66 (11.8)
Female sex (%)	2714 (53.6)	1042 (55.6)	1382 (52.7)	290 (51.8)
Mean haemoglobin concentration (g/dL) (SD)				
Males	13.2 (2.8)	11.4 (3.4)	14.5 (1.2)†	-
Females	12.0 (1.8)	10.5 (1.5)	13.2 (0.9)†	-
Median C reactive protein concentration‡ (mg/L) (IQR)	2.3 (1.24.3)	2.3 (1.3, 4.5)	2.3 (1.2, 4.2)†	2.3 (1.3, 4.7)
HIV positive (%)	1047 (20.7)	534 (28.5)	472 (18.0)†	41 (7.3)†
Previous angina (%)	456 (9.0)	183 (9.8)	250 (9.5)	23 (4.1)†
Previous stroke (%)	149 (2.9)	63 (3.4)	74 (2.8)	12 (2.1)
Hypertension (%)	3145 (62.2)	1151 (61.4)	1702 (64.9)†	292 (52.1)†
Diabetes mellitus (%)	559 (11.0)	229 (12.2)	286 (10.9)	44 (7.9)†
Chronic bronchitis (%)	28 (0.6)	15 (0.8)	10 (0.4)	3 (0.5)
Highest level of education (%)‡				
No education	2306 (45.7)	901 (48.2)	1151 (44.0)†	254 (45.6)†
Year 1 – 7 (primary)	1716 (34.0)	640 (34.2)	915 (35.0)†	161 (28.9)†
Year 8 – 11 (secondary)	574 (11.4)	206 (11.0)	305 (11.7)†	63 (11.3)†
Year 12+ (secondary and above)	446 (8.8)	123 (6.6)	244 (9.3)†	79 (14.2)†
Mean 5 m walk speed (m/s) (SD)				
Males	0.65 (0.33)	0.66 (0.32)	0.68 (0.29)	0.51 (0.44)†
Females	0.62 (0.33)	0.64 (0.32)	0.63 (0.32)	0.45 (0.35)†
Mean maximum grip strength (kg) (SD)				
Males	29.5 (12.7)	28.9 (11.1)	31.5 (11.8)†	22.6 (17.6)†
Females	22.0 (9.5)	21.7 (8.7)	23.1 (9.0)†	17.4 (12.8)†
Cognitive z-score (SD)‡	0 (1)	−0.05 (0.99)	0.04 (0.99)†	−0.04 (1.06)
Mean body mass index (kg/m ^2^) (SD)				
Males	24.9 (5.5)	24.4 (5.3)	25.3 (5.5)†	24.5 (6.3)
Females	29.3 (7.5)	28.9 (8.0)	29.6 (7.1)†	28.9 (6.8)
≥1 basic ADL impairment (%)	476 (9.4)	188 (10.0)	223 (8.5)	65 (11.6)

*Haemoglobin (Hb) <13.0 g/dL for men, <12.0 g/dL for women.

†P<0.05 compared with individuals with anaemia.

‡Cognitive score data for 4927 individuals (4404 in the analysis sample); C reactive protein data for 4302 individuals (4124 in the analysis sample); education data for 5042 individuals (4485 in the analysis sample).

ADLs, activities of daily living.

Compared with the analysis population, the 560 HAALSI participants without haemoglobin concentrations were of similar age and sex distribution to those with haemoglobin measurements but had less comorbidity, lower grip strength and lower walk speed than those with haemoglobin concentrations available. A total of 833/2075 (40.1%) men and 1042/2424 (43.0%) women with haemoglobin measurements were anaemic. Anaemia was of moderate severity in 201/2075 (9.7%) men and 481/2424 (19.8%) women and was severe in 38/2075 (1.8%) men and 67/2424 (2.8%) women.

**Figure 1:**
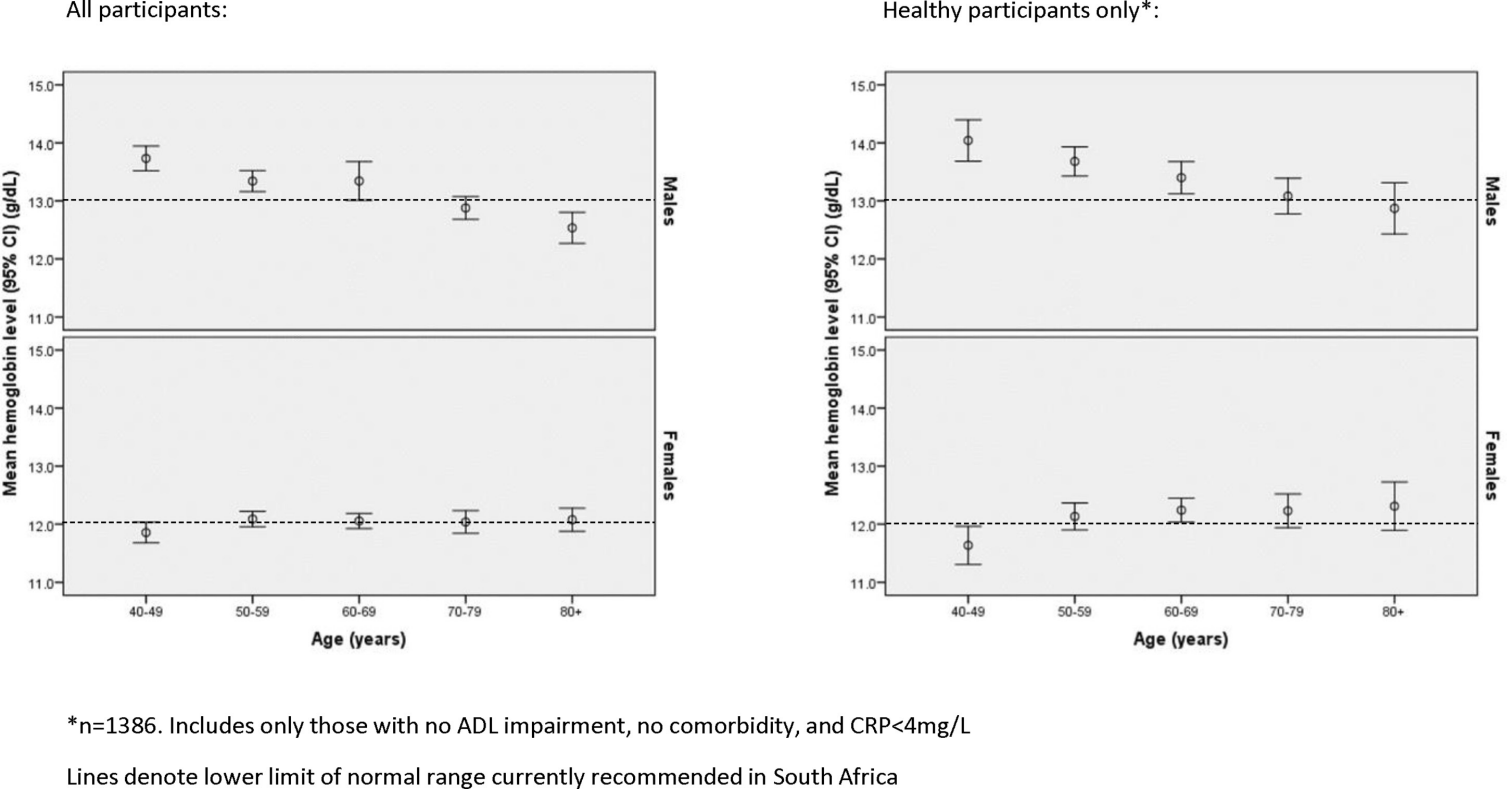
Relationship between age and haemoglobin concentration. ADL, activities of daily living; CRP, C reactive protein.

Figure 1 shows the relationship between age and haemoglobin concentration separately for men and women. Haemoglobin concentration was inversely associated with age for men (table 2; r=−0.121; p<0.001), both when considering all men and when limiting the sample to only healthy men. In contrast, no relationship was seen between age and haemoglobin concentration when data from all women were analysed (table 2; r=0.021; p=0.30). When data from healthy women were analysed, however, haemoglobin was positively correlated with age (r=0.10; p=0.007).

**Table 2:** Bivariate relationships between haemoglobin concentrations, physical and cognitive performance and age as a continuous variable (n=4499)

	Men		Women	
	Correlation coefficient	P values	Correlation coefficient	P values
Walk speed*	0.057	0.009	0.011	0.60
Grip strength*	0.161	<0.001	0.076	<0.001
Cognitive z-score	0.047	0.04	0.027	0.19
Age	−0.121	<0.001	0.021	0.30
Age; healthy only†	−0.189	<0.001	0.100	0.007

Pearsons correlation coefficient except for *Spearman’s rho.

†Excluding those with comorbid disease, impairment of activities of daily living or C reactive protein ≥4 mg/L.

**Figure 2:**
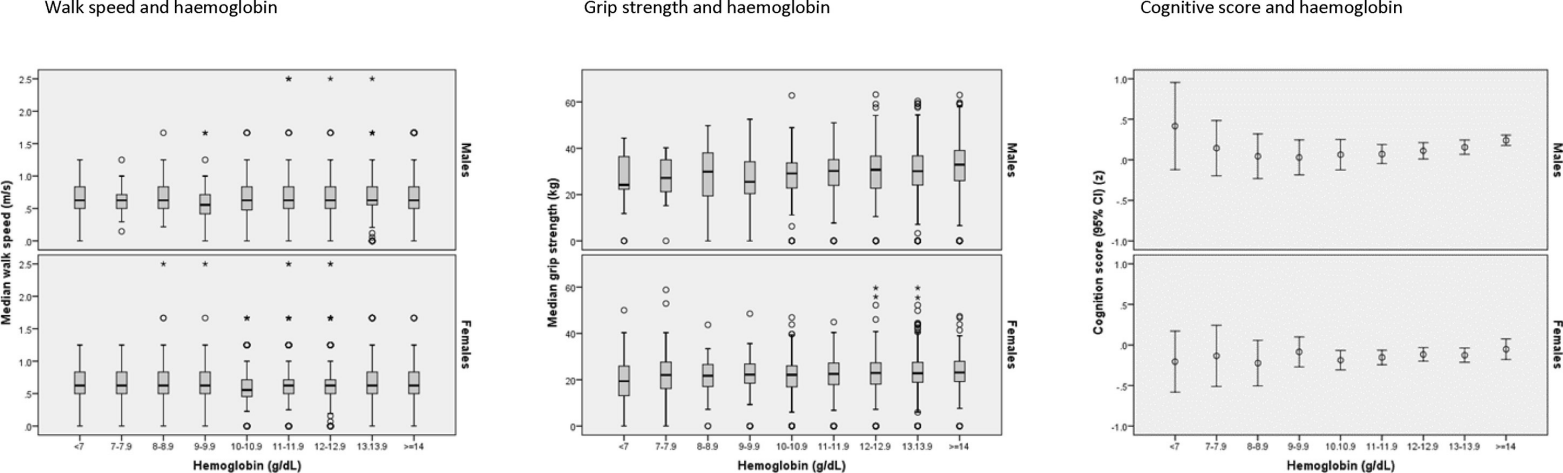
Relationship between haemoglobin concentration and measures of physical and cognitive function.

Figure 2 shows the association between haemoglobin concentration and each of grip strength, walk speed and cognitive z-score, depicted separately for men and women; table 2 shows the correlations between haemoglobin concentrations and each of measures of physical and cognitive performance and age. Haemoglobin concentration was negatively correlated with age, both in all men and the subgroup of healthy men; the correlation was significant for healthy women but not when all women were considered. Haemoglobin and grip strength were positively correlated in both men and women; weakly positive correlations were seen between haemoglobin and each of walk speed and latent cognitive z-score for men but not for women (figure 2 and table 2).

Mean haemoglobin concentrations varied little between those with and without specific comorbidities except that mean haemoglobin concentrations were lower in people who were HIV positive versus HIV negative (13.0 vs 13.3 g/dL; p=0.02 in men; 11.6 vs 12.2 g/dL; p<0.001 in women) and men with versus without chronic bronchitis (online supplementary table 1). There was a weak but statistically significant inverse association between C reactive protein concentration and haemoglobin concentration in men but not in women (Spearman’s rho=−0.09, p<0.001 in men; rho=−0.002, p=0.93 in women).

Table 3 shows the results of multivariable-adjusted regression analyses. Haemoglobin concentration was independently associated with grip strength in women when covariates were included in the model (B=0.391; 95% CI 0.177 to 0.605), but this association was not statistically significant in men (B=0.266; 95% CI −0.019 to 0.552). Haemoglobin was not independently associated with walk speed or cognitive z-scores in men or women when other covariates were included in the model (table 3). Age was inversely associated with haemoglobin after adjustment for comorbidities and CRP concentration for men, with haemoglobin levels being 0.30 g/dL (95% CI 0.20 to 0.40) lower, on average, for each decade of age. In contrast, haemoglobin was not associated with age among women (B=−0.01; 95% CI −0.07 to 0.05).

**Table 3:** Association between haemoglobin and physical and cognitive measures after adjustment for covariates

	Grip strength*				Walk speed*				Cognition score†			
	Men (n=1882)		Women (n=2239)		Men (n=1882)		Women (n=2239)		Men (n=1840)		Women (n=2187)	
	B	P values	B	P values	B	P values	B	P values	B	P values	B	P values
Haemoglobin	0.266	0.07	0.391	<0.001	−0.001	0.71	−0.002	0.51	0.003	0.71	0.014	0.13
Diabetes	−0.241	0.80	−0.377	0.52	−0.075	0.001	−0.018	0.35	0.043	0.48	−0.087	0.08
Hypertension	1.816	0.004	0.683	0.12	−0.008	0.63	−0.010	0.51	0.162	<0.001	0.050	0.16
Angina	0.570	0.61	0.300	0.63	0.013	0.64	−0.016	0.45	0.031	0.65	0.128	0.01
Stroke	−5.938	<0.001	−3.857	<0.001	−0.170	<0.001	−0.215	<0.001	−0.432	<0.001	−0.166	0.08
HIV	0.921	0.24	0.163	0.75	0.040	0.03	0.027	0.12	0.183	<0.001	−0.030	0.46
Chronic bronchitis	−1.819	0.57	2.792	0.39	0.081	0.37	−0.026	0.79	−0.122	0.57	−0.387	0.12
Log _e_CRP	−0.674	0.03	−0.033	0.88	−0.013	0.09	−0.023	0.002	−0.016	0.40	0.054	0.002
Highest year education												
None									Referent	–	Referent	–
1–7									0.497	<0.001	0.754	<0.001
8–11									0.742	<0.001	1.047	<0.001
12+									1.024	<0.001	1.316	<0.001
Age	−0.338	<0.001	−0.273	<0.001	−0.004	<0.001	−0.007	<0.001	−0.013	<0.001	−0.026	<0.001

All models adjusted for all listed variables.

*Generalised linear model using Tweedie distribution, power 1.2 with identity link.

†Linear regression.

The subgroup who were HIV positive showed the same pattern of associations as the whole cohort: haemoglobin and grip strength were positively correlated for men and women (online supplementary table 2), and haemoglobin was weakly positively correlated with walk speed in men only. In multivariable-adjusted analyses, haemoglobin was independently associated with grip strength only for women (online supplementary table 3).

## Discussion

In this population-based study of men and women aged 40 years and over in rural South Africa, we found a high prevalence of anaemia according to the current WHO thresholds, and over twice the prevalence seen in the South African National Health and Nutrition Examination Survey (SANHANES) survey of the general South African population.^10^ The prevalence of anaemia increased with age among men only; this decrease in haemoglobin concentrations with advancing decade of age in men was not explained by our measures of comorbid disease, inflammation or activity limitation. In contrast, women did not exhibit lower haemoglobin concentrations at older ages in this study. After adjusting for age, comorbid disease and chronic inflammation, haemoglobin levels were not significantly associated with grip strength in men, or with walking speed or cognitive function in men or women. Similar patterns were found in the subgroup of middle-aged and older adults who were HIV positive.

Many diseases that impair physical and cognitive performance are known to depress haemoglobin concentrations,^19–21^ and multimorbid patients are also more likely to have poor nutritional intake or chronic inflammation as causes of anaemia. Any association between haemoglobin levels and impaired physical or cognitive function might therefore be confounded by comorbid disease. Previous work has shown associations between lower haemoglobin and impaired performance on a number of tests, including grip strength, knee extensor strength, walk speed, timed up and go and the Short Physical Performance Battery.^7 22^ Interestingly, no association was seen between anaemia and physical performance in a study of older patients with a range of medical conditions discharged from hospital in Belgium.^23^ It is unclear why our results should differ from those seen in previous work, but our ability to adjust for both chronic inflammation and a more extensive range of comorbidities than previous work may partly explain the differences. Most previous work has been conducted in HICs, in predominantly white populations. One exception was the US NHANES III study, which suggested that African-Americans have lower haemoglobin concentrations than individuals of European ancestry and that the threshold at which haemoglobin starts to associate with increased mortality is lower in African-Americans.^24^ At present, it is not clear if differences between populations in patterns of associations between haemoglobin levels and outcomes are due to differences in wealth, diet, causes of anaemia, genetic factors, availability of healthcare or other unmeasured factors. Only cross-country, cross-population studies will be able to address this question.

A further potential explanation, and limitation of the present study, is selection bias. Individuals with very poor physical or cognitive function might be expected to have a lower chance of participating in the study interview and data collection process, although their non-participation makes this difficult to prove. If this occurred, it would have diluted any observed relationship between haemoglobin and physical or cognitive performance measures in this study. This may be an issue in rural South Africa where access to formal social support and to healthcare is limited and cannot thus mitigate the impact of disease and functional decline. If lower haemoglobin is associated with a higher risk of death, those older adults with the lowest haemoglobin would be less likely to survive to take part in HAALSI, further diluting any association. In addition, the content of the cognitive tests we used focused on verbal memory and orientation. We did not have direct measures of executive function, attention and processing speed were more prominent components of the tests.^5^ Finally, although our latent cognitive function outcome variable does not translate to a clinically defined outcome of cognitive impairment or dementia, it captures the full range of cognitive function from impaired to high-performing, as was the intent of the study cognitive battery.

Controversy continues as to whether age per se is a cause of lower haemoglobin concentrations, and therefore whether different normal ranges are appropriate for different ages. Bone marrow cellularity declines with age, suggesting a loss of capacity to produce new red cells. If red cell life is shortened (eg, by haemolysis) or red cells are lost (eg, by bleeding), older bone marrow may lack the capacity to respond adequately to replace lost cells. While some observational studies show that advanced age is associated with lower haemoglobin concentrations,^7 25–27^ haemoglobin concentrations are maintained in some groups of very old, very fit individuals.^28^ Age-related bone marrow dysfunction alone is unlikely to be sufficient to explain the associated between age and lower haemoglobin levels, and other diseases (that often accompany ageing) may be necessary to explain this association.^29^

While our results agree with these previous findings for men, haemoglobin did not decline with increasing age cohort in women. Indeed, haemoglobin concentrations in the youngest group of healthy women were lower than those seen in older groups, which may reflect the impact of menstrual blood loss in younger women. This patterning by sex is similar to that shown in the general South African population, although the prevalence of anaemia we observed was twice that seen in the SANHANES cohort, which included a younger sample.^10^ The reasons for the difference in the age–haemoglobin relationship between men and women remains unclear as it persisted after adjustment for comorbidities and CRP. It is possible that age-related changes in testosterone could explain some of this difference.^30^

Our results do not support a change in the normal range of haemoglobin with age in women. The issue is more complex for men, but it is striking that over half the men aged 80 years or over who did not have comorbid disease or elevated CRP fulfilled the current definition for anaemia. Labelling such a large number of people with anaemia carries significant consequences for investigation and management, both at the level of the individual and for health systems.

Our analysis has a number of strengths, principally our ability to study a large sample representative of the middle-aged and older men and women residing in this rural area of South Africa. Our ability to adjust for chronic inflammation and for comorbid disease further strengthens this analysis, as does the use of cognitive tests tailored to the cultural context within which HAALSI was conducted. A number of limitations also bear consideration. The data are cross-sectional, and so we are unable to test how haemoglobin levels might predict future changes in physical or cognitive performance. HAALSI did not collect blood samples that would allow for measurement of red cell indices, and data are not available on haematinic concentrations, creatinine or serology for other potential causes of anaemia. Some diagnoses are unlikely to be common in this population; for example, the prevalence of the sickle-cell allele is low (<3%).^31^ The Agincourt HDSS is sited in an area prone to malaria, which may contribute to anaemia, and the prevalence of iron deficiency in South Africa is high, as noted earlier, although the underlying causes of such deficiency cannot be ascertained from the current study. We cannot exclude the possibility that undiagnosed illnesses were present, such as cancer or chronic infections or infestations; this is particularly likely to be an issue in this underserved rural population who lack access to comprehensive, proactive primary healthcare. Conversely, some diagnoses, particularly hypertension and diabetes, were made on the basis of readings on a single day in the cross-sectional HAALSI data, with the potential to overdiagnose these conditions. Although our sample size was adequate to detect modest correlations (over 80% power to detect r=0.04 at an alpha of 0.05), weak associations that might still be of interest at the population level may not have been detectable. Relating the complex distribution of grip strength and walk speed to unit changes in haemoglobin is difficult, and our use of a Tweedie distribution in these analyses did not permit us to derive a simple measure of difference in haemoglobin per unit change in outcome measure for these outcomes.

Perhaps most importantly, this research raises the question of what action should be triggered by low haemoglobin concentrations in middle-aged and older adults in rural South Africa? Superficially, anaemia appears to be an attractive intervention target to improve the health of older people in LMICs: interventions such as iron or vitamin replacement are inexpensive, relatively safe and could be scaled to population level. However, the outcomes that older people value most are physical function and quality of life.^32^ Currently, there is no evidence to show that intervening to increase haemoglobin concentrations in mild anaemia improves symptoms, or physical or cognitive function in older people. Randomised controlled trials are needed to test whether strategies to treat anaemia improve physical function and quality of life, rather than merely changing haemoglobin concentrations.^33^

In conclusion, our results suggest that although anaemia is very common in middle-aged and older people living in rural South Africa, haemoglobin levels are not associated with most measures of physical and cognitive function that are important to older people. Further longitudinal research is required to understand the aetiology and consequences of anaemia in this population, as well as to design efficient systems for the investigation and management of anaemia in this population. It may be, however, that the best way to improve function, quality of life, and even lifespan in rural South Africans with anaemia is to focus on effective management of the comorbidities that contribute to anaemia, rather than the anaemia itself.

**What is already known on this subject**

- Haemoglobin levels are lower at older ages in high-income countries, but it is unclear if this is due to age itself or coexistent disease.
- In high-income countries, haemoglobin levels are inversely associated with physical and cognitive performance in older people.
- The relationships between haemoglobin and age and physical and cognitive performance are unclear in middle-aged and older people in lower and middle-income countries.

**What this study adds**

- The prevalence of anaemia was 40% in this rural South African population of people aged over 40 years old; most anaemia was in the mild category.
- Prevalence was higher than that seen in similar aged populations in high-income countries.
- In contrast with many studies in high-income countries, haemoglobin concentration was not independently associated with physical or cognitive function
- Our findings suggest that interventions to improve haemoglobin, which are common practice in many high-income countries, might not lead to improvements in physical or cognitive function, even for moderate or severe anaemia.
- Our findings need to be replicated in other lower income and middle-income country contexts.
