# Supplementary material for: Cross-sectional relationship between haemoglobin concentration and measures of physical and cognitive function in an older rural South African population
Source: J Epidemiol Community Health. 2018 Apr 21;72(9):796–802. doi: 10.1136/jech-2018-210449 (PMC6109255; doi:10.1136/jech-2018-210449)
Supplement: Supplementary data [file jech-2018-210449supp001.pdf]

**Supplementary Information**

*Physical and cognitive performance measures*

To measure walk speed, participants were asked to walk a 2.5m course twice, with the time taken timed to the nearest 0.1s. The course was marked out on flat, obstacle-free ground in or around the participant's home. Participants were allowed to use any usual walking aids but no human assistance was permitted; participants started the course from a stationary standing position. The second walk was conducted on a course that was the reverse of the first course. Participants who were unable to conduct the walk test were assigned a walk speed of 0m/s; those who declined to participate were omitted from analysis. The mean speed of the two walks was the outcome measure.

Grip strength was measured twice in both hands, using a Smedley digital dynamometer (12-0286). Testing was conducted with participants in a seated position, with the arm under evaluation held at 90 degrees of elbow flexion. For this analysis, the maximum reading achieved was used. No participant declined to participate in grip strength testing; participants who were unable to perform grip strength testing because of pain or deformity in both hands were assigned a grip strength of 0kg.

Cognitive performance was assessed in the main study interview with a cognitive battery adapted for language, cultural, and educational appropriateness from validated measures used in the U.S. Health and Retirement Study. The cognitive battery assessed time orientation (ability to correctly state the current day, month, year, and South African president; one point for each item), immediate and delayed recall of ten words that were read aloud by the interviewer (one point for each correct word recalled, for a total of 20 points), and numeracy (ability to correctly count from 1 to 20, and ability to complete the numerical pattern of "2, 4, 6…" with the correct response of "8"; one point for each item). A total of 26 points were therefore awarded for the cognitive battery. Some components of the test battery have previously been used in widely-used cognitive test scores including the

Abbreviated Mental Test Score and the Mini-Mental Status Examination. Scores on the immediate and delayed recall items have previously been associated with risks of dementia and all-cause mortality in older adults, and the two numeracy items reflect an ability-based outcome of education (i.e. numeracy) that has direct relevance for daily functioning and well-being. The cognitive battery was translated and back-translated from English to the local Shangaan language and was pilot tested prior to the HAALSI study with a community-based advisory panel of 90 local adults aged 40 and over, none of whom were part of the later cohort enrolment.

A z-standardized, general latent cognitive factor was derived from the individual cognitive tests using confirmatory factor analysis, the methods and results of which are described in detail elsewhere [1]. The latent cognitive function z-score should be interpreted in the same way as any z-score (i.e. the mean is 0 and the standard deviation is 1), and conceptually it makes the assumption that scores on each individual cognitive test are reflective of a general underlying cognitive ability. This method improves upon the use of a composite total score (i.e. summed cognitive battery score out of 26 points), as it reduces measurement error that may be present in any single cognitive item by only utilizing the covariation between items in generating the latent cognitive z-score, and it differentially weights the individual items according to their contribution to the latent cognitive z-score. For example, a composite total score would assign equal weight to knowledge of the current day (one point) with the ability to remember an additional word from the word recall list (one point), although these two cognitive tasks may vary in difficulty and may have differential relationships to functional outcomes and future health risk. The confirmatory factor analysis model was of good fit to the data, with a root mean square error of approximation of 0.032 (95% CI: 0.025, 0.039), comparative fit index of 0.998, and Tucker-Lewis index of 0.997 [1].

*Covariates*

Age was ascertained by self-report from participants, rounded to the nearest year. Sex was ascertained by self-report. Educational status was based on the highest year of school attended; none,

years 1-7, denoting some primary-level education; years 8-11, denoting some secondary education, or years 12 and above, denoting completed secondary education or post-secondary education. Self-reported angina, chronic bronchitis and stroke were ascertained by a series of questions asking whether a physician had ever diagnosed these conditions or whether participants were on treatment for these conditions. In addition, the Rose angina questionnaire was administered to ascertain whether angina symptoms were present [2]. Diabetes mellitus was ascertained by asking if a participant was taking medication for the condition, if the participant had ever been told that they had diabetes, or by a random capillary glucose concentration >11.0mmol/L obtained as part of the HAALSI survey. Blood pressure was measured in the seated position; three readings were taken and the mean of the second and third reading was used as the measure of blood pressure. Hypertension was ascertained by asking if the participant was taking medication for hypertension at the time of interview, or by a systolic blood pressure >140mmHg, or diastolic blood pressure >90mmHg at the time of interview.

C-reactive protein was measured from dried blood spots using a high-sensitivity sandwich ELISA method as described previously [3]. The lower limit of sensitivity for the assay was 0.05mg/L. HIV status was ascertained by analysis of dried blood spot samples using the Vironostika Uniform 11 assay (Biomerieux, France).

References:

[1] Kobayashi LC, Glymour MM, Kahn K et al. Childhood deprivation and later-life cognitive function in a population-based study of older rural South Africans. *Soc Sci Med* 2017;190:20-8.

[2] Rose GA, Blackburn H. Cardiovascular Survey Methods, 1968: Geneva. WHO

[3] McDade TW, Burhop J, Dohnal J. High-sensitivity enzyme immunoassay for C-reactive protein in dried blood spots. *Clin Chem* 2004;50:652-4.

**Supplementary Table 1. Mean haemoglobin levels according to the presence of individual comorbidities**

| | Hb concentration (g/dL) (SD) - men | | | Hb concentration (g/dL) (SD) - women | | |
|---|---|---|---|---|---|---|
| *Comorbidity* | *Present* | *Absent* | *P** | *Present* | *Absent* | *P** |
| Diabetes | 13.0 (2.2) | 13.3 (2.8) | 0.22 | 11.9 (2.0) | 12.1 (1.8) | 0.07 |
| Hypertension | 13.3 (2.0) | 13.2 (3.6) | 0.71 | 12.1 (1.8) | 11.9 (1.8) | 0.08 |
| Angina | 12.9 (2.1) | 13.3 (2.8) | 0.07 | 12.1 (1.9) | 12.0 (1.8) | 0.55 |
| Stroke | 12.7 (2.0) | 13.3 (2.8) | 0.13 | 11.7 (2.0) | 12.0 (1.8) | 0.16 |
| HIV | 13.0 (4.5) | 13.3 (2.0) | 0.02 | 11.6 (1.6) | 12.2 (1.8) | <0.001 |
| Chronic bronchitis | 11.8 (2.4) | 13.3 (2.8) | 0.04 | 12.2 (1.2) | 12.0 (1.8) | 0.83 |

*Students t-test

**Supplementary Table 2**
**Bivariate relationships between covariates and haemoglobin concentrations in HIV positive individuals (n=1006)**

| | M | | F | |
|---|---|---|---|---|
| | r | p | r | p |
| Walk speed* | 0.091 | 0.05 | 0.051 | 0.23 |
| Grip strength* | 0.160 | 0.001 | 0.114 | 0.008 |
| Cognitive z score | -0.010 | 0.83 | 0.041 | 0.35 |
| Age | -0.023 | 0.62 | -0.008 | 0.85 |

Pearsons chi-squared exc for *Spearmans rho

**Supplementary Table 3**

**Association between haemoglobin and physical and cognitive measures after adjustment for covariates - HIV positive participants only (n=1006)**

| | | Grip strength* | | | | Walk speed* | | | | Cognition score** | | | |
|---|---|---|---|---|---|---|---|---|---|---|---|---|---|
| | | Men (n=443) | | Women (n=515) | | Men (n=443) | | Women (n=515) | | Men (n=437) | | Women (n=507) | |
| | | B | p | B | p | B | p | B | p | B | P | B | p |
| Haemoglobin | | 0.200 | 0.40 | 0.565 | 0.02 | 0.001 | 0.67 | -0.003 | 0.73 | 0.000 | 0.97 | 0.006 | 0.78 |
| Diabetes | | 0.480 | 0.84 | 0.336 | 0.83 | -0.035 | 0.50 | -0.009 | 0.86 | 0.083 | 0.59 | -0.093 | 0.46 |
| Hypertension | | 1.353 | 0.29 | 1.286 | 0.12 | -0.022 | 0.44 | 0.020 | 0.46 | 0.040 | 0.62 | 0.233 | 0.001 |
| Angina | | 1.426 | 0.58 | 0.124 | 0.93 | 0.080 | 0.19 | -0.070 | 0.11 | 0.153 | 0.34 | 0.128 | 0.29 |
| Stroke | | -5.829 | 0.07 | -4.798 | 0.11 | -0.098 | 0.19 | -0.254 | 0.006 | -0.539 | 0.02 | -0.752 | 0.03 |
| Chronic bronchitis | | -5.302 | 0.37 | 3.622 | 0.58 | 0.065 | 0.68 | -0.218 | 0.18 | -0.940 | 0.02 | -0.534 | 0.32 |
| $Log_e$CRP | | 0.375 | 0.57 | -0.110 | 0.80 | -0.030 | 0.06 | -0.003 | 0.82 | -0.014 | 0.74 | -0.023 | 0.53 |
| Highest year of education | None | | | | | | | | | Referent | - | Referent | - |
| | 1-7 | | | | | | | | | 0.470 | <0.001 | 0.741 | <0.001 |
| | 8-11 | | | | | | | | | 0.680 | <0.001 | 1.139 | <0.001 |
| | 12+ | | | | | | | | | 1.040 | <0.001 | 1.219 | <0.001 |
| Age | | -0.305 | <0.001 | -0.274 | <0.001 | -0.003 | 0.04 | -0.002 | 0.07 | -0.011 | 0.01 | -0.016 | <0.001 |

*Generalised linear model using Tweedie distribution, power 1.2 with identity link

** Linear regression

All models adjusted for all listed variables
